# Validation of the Dominican system for measuring early childhood development

**DOI:** 10.12688/f1000research.128657.2

**Published:** 2024-01-29

**Authors:** Laura V. Sánchez-Vincitore, María Angélica Alonso Pellerano, María Elena Valdez, Angie Sabrina Jiménez, Carlos B Ruiz-Matuk, Arachu Castro, Felipe Díaz, Daniel Cubilla-Bonnetier

**Affiliations:** 1 Universidad Iberoamericana (Unibe), Santo Domingo, Dominican Republic; 2 Instituto Nacional de Atención Integral a la Primera Infancia (INAIPI), Santo Domingo, Dominican Republic; 3 Tulane University, New Orleans, USA; 4 United Nations International Children's Emergency Fund (UNICEF-DR), Santo Domingo, Dominican Republic

**Keywords:** Early childhood development, Dominican Republic, Monitoring, Screening

## Abstract

**Background:**

The purpose of the study was to determine the psychometric properties of the Dominican System for Measuring Early Childhood Development (SIMEDID, for its Spanish acronym), to adjust the sequence of item presentation, and to provide age-standardized norms for each item, to enable policy and program managers to make decisions based on specific and structured data.

**Methods:**

After approval from an ethics committee, a total of 948 children from 0 to 60 months participated in this study. Participants were evaluated on four early childhood development domains (gross motor, fine motor, language development, and socio-emotional development). The data were collected from November 2021 to February 2022, either at early childhood care centers or at home, using mobile devices that guided the evaluators through the screener. Data were later synced to a global database. Psychometric properties were calculated using Cronbach’s alpha and split-half parallel reliability. For reorganizing item presentation and to obtain age-standardized norms, we conducted a logistic regression analysis for each item on dependent variable item success, and independent variable age.

**Results:**

The instrument showed excellent reliability and additional evidence of validity. The item presentation order was rearranged according to the probability of item success progression. In addition, the study characterized the expected evolution of item success probability across participants’ age.

**Conclusions:**

SIMEDID is a valid and reliable instrument for depicting childhood development in national evaluations. Its integration with electronic platforms for national monitoring represents a cost-effective, time-efficient screening tool adapted to the Dominican sociocultural context. This represents a promising tool to strengthen strategies that support early childhood development.

## Introduction

The Dominican Republic confirmed its commitment to achieving the United Nation’s sustainable development goal 4.2 for 2030 to guarantee that children are ready for primary education by offering quality early childhood services, including care and early education ( United Nations, 2015). One of this goal’s indicators is “[the] proportion of children aged 24-59 months who are developmentally on track in health, learning, and psychosocial well-being, by sex” ( UNESCO UIS, n.d., para. 1). Although this indicator is conceptually straightforward, and numerous efforts have been conducted to establish a global methodology for measuring it, some challenges associated with determining cut-off points for early childhood development remain ( Daelmans *et al.,* 2017; Richter *et al.,* 2017).

First, high-income countries’ screening tools might translate poorly to low- and middle-income countries. This poses the risk of either underestimating or overestimating childhood development, which in turn precludes making accurate, evidence-based decisions regarding early childhood interventions and resource allocation ( Gladstone *et al.,* 2008; Sabanathan *et al.,* 2015). The second challenge is the need for more funding for monitoring systems in low- and middle-income countries, which translates into needing more sufficiently qualified personnel to conduct periodic childhood development screenings ( Lokuketagoda *et al.,* 2016). And third, there is a critical need to obtain a large pool of data from developing children to identify those at risk for developmental delay ( Lokuketagoda *et al.,* 2016), which is particularly challenging in low- and middle-income countries ( Richter *et al.,* 2017).

In Latin America and the Caribbean, childhood development measurement has attracted attention, evidenced by the creation of the Regional Network for Measuring Childhood Development (REMDI) ( Interamerican Dialogue, 2020). This international network of specialists is dedicated to promoting national measurements of childhood development to obtain data for decision-making and comparison between and within countries. Since the year 2000, the Dominican Republic has collected data on childhood development by participating in a series of Multiple Indicator Cluster Surveys (MICS), a household survey methodology designed by UNICEF that analyzes the situation of women and children across the world. The instrument collects data on children’s health, education, protection, and environment (such as sanitation), among other variables. The Dominican Republic participated in MICS2 ( Molina Achécar and Polanco, 2001), MICS5 ( ONE and UNICEF, 2016), and MICS6 ( ONE and UNICEF, 2021) survey rounds with an evolving early childhood development measurement ( Loizillon *et al.,* 2017).

The latest data from 2019 reported that 87.1% of Dominican children meet the minimum development indicators. Data generated by MICS have been useful for guiding the advocacy and system-strengthening plans of early childhood development and children’s rights institutions, including sustainable investments in the multi-year governmental planning for 2020-2024. In addition, the MICS data have been used to create predictive models that quantify the impact of multiple sociodemographic and psychosocial factors in childhood development ( Sánchez-Vincitore and Castro, 2022).

Household surveys provide useful information on general trends in childhood development, but they are not comprehensive enough to assess development in its various dimensions or sensitive enough to generate alerts to detect developmental delays. These limitations highlight the need for specific child development screening tools that, although quick and cost-effective in the application, have adequate psychometric properties.

Many private, informal, and some public initiatives have been conducted in the Dominican Republic to provide early childhood services. However, until 2019 there were no standardized instruments to measure the impact of such efforts. In 2019, Sánchez-Vincitore *et al.* (2019) initiated the validation of the Dominican adaptation of the Malawi Developmental Assessment Tool (MDAT) ( Gladstone *et al.,* 2008, 2010). The MDAT is a childhood development screener in which an evaluator observes the behavior of a child in four different domains: gross motor, fine motor, language, and socio-emotional development. One of the advantages of MDAT is that it optimizes test application time by only presenting items that correspond to the child’s expected evolutionary stage according to the child’s age. Therefore, providing a more precise item order is crucial to prevent bias in obtaining total scores. The adaptation of the MDAT to the Dominican Republic (MDAT-DR) ( Sánchez-Vincitore *et al.*, 2019) presented good psychometric properties. However, some limitations had to be considered before upscaling it as a national surveillance tool. First, as a preliminary pilot implementation within the academic context, research assistants with vast experience and training in data collection administered the instrument, which is unlikely in naturalistic environments. Second, the MDAT-DR adaptation had a small sample size (N = 42), meaning there was no national representation. Therefore, age-standardized norms were not obtained for each item, threatening item presentation order. Finally, the study evaluated children up to 24 months, limiting the age range for which data were available.

To overcome these limitations, the National Institute for Early Childhood Comprehensive Care (INAIPI, for its acronym in Spanish), established as a national state institution in 2013 to ensure quality comprehensive care services to children aged 0 to five and their families, collaborated with the Universidad Iberoamericana (UNIBE) and the United Nations Children’s Fund (UNICEF) to develop the Dominican System for Measuring Early Childhood Development (SIMEDID, for its acronym in Spanish). To create SIMEDID, the team re-analyzed the content and structure of the MDAT-DR ( Sánchez-Vincitore *et al.,* 2019) and integrated other items from international and national instruments ( Alonso *et al.,* 2022). The research team obtained an expert panel’s consensus before collecting data to guarantee the instrument’s construct definition ( Sireci and Sukin, 2013). The expert panel consisted of a group of professionals who represent Dominican institutions that provide early childhood services, including the Early Childhood Education Department and the Special Education Department from the Ministry of Education; the Ministry of Health; the National Health Services (SNS for its Spanish acronym); the National Council for Childhood and Adolescence (CONANI for its Spanish acronym); and the National Council for Disabilities (CONADIS for its Spanish acronym). The experts had the opportunity to revise each item and their definitions.

The team adopted all four of MDAT-DR’s dimensions—including fine motor skills, gross motor skills, social and emotional development, and language development—updated them for relevance, created a progressive item list for each developmental dimension through expert input, and revised the instrument twice based on surveys among educators and facilitators for clarity. Next, the team conducted an initial pilot of SIMEDID aimed to assess its application conditions, digital platform functionality, internal consistency, and user experience ( INAIPI, 2020; Sánchez-Vincitore, 2020). The pilot study included 100 children aged 45 days to 5 years who were receiving INAIPI services. The study confirmed the platform’s offline functionality, speed, and user-friendly interface, along with the willingness of educators and animators to participate. In addition, the instrument showed good internal consistency.

SIMEDID is an electronic platform that hosts an early childhood development screener. This platform allows data collection through mobile devices and connects to INAIPI’s servers as part of its monitoring and evaluation system. The mobile application extracts sociodemographic information from the server, configuring individual evaluations for each child’s age. As a result, INAIPI personnel already in the field can administer the early childhood development screener time-efficiently with little training. Once the assessment is over and the device connects to the internet, the data sync to the server—reducing the risk of losing the data.

We conducted this study to validate SIMEDID, with three aims: (1) To determine the psychometric properties of SIMEDID; (2) To adjust the sequence of item presentation according to developmental milestones obtained from data from a large sample; (3) To provide age-standardized norms for each item.

## Methods

### Ethical statement

The Universidad Iberoamericana’s ethics committee approved this study (CEI2021-3). Written informed consent was obtained from the participant’s parents or guardians before participation in the study.

### Study design

This is a cross-sectional, non-experimental, and descriptive study that evaluated children who receive services at INAIPI.

### Setting

Data collection occurred in Santo Domingo, Dominican Republic, from November 1 ^st^, 2021, to February 17 ^th^, 2022. INAIPI participants who attended Comprehensive Care Centers for Early Childhood (CAIPIs, for its Spanish acronym) were assessed at their centers. In contrast, those who participated in Comprehensive Care Centers for Children and the Family (CAFI, for its Spanish acronym) received community and family-based services at their home, which is where children were assessed. The instrument was applied during regular service hours. The evaluation personnel consisted of 20 educational agents (who work at CAIPIs) and 20 community agents (who work at CAFIs).

### Participants

An intentional sample of 948 children who live in Santo Domingo was selected from the INAIPI’s System of Information and Management for Early Childhood (SIGEPI, for its Spanish acronym) according to their age and type of service received (CAIPI or CAFI, which were kept proportional to the actual service: 36% and 64%, respectively). The inclusion criterion was to be beneficiaries of INAIPI. Participants were 428 girls (45.1%) and 520 boys (54.9%).

### Instruments

*Sociodemographic variables:* This set of questions addressed general demographic variables: sex assigned at birth (male and female), age (in days at the moment of evaluation), and type of service (CAIPI vs CAFI). These variables, obtained directly from SIGEPI, determine the starting item of SIMEDID ’s subscales.

*SIMEDID:* This electronic instrument assesses childhood development in four development areas: gross motor, fine motor, language development, and social development. The assessment tool requires an observation of the child’s behavior, either by the evaluator, parent, or the person in charge of the child at a given time. If the child does not show the expected behavior at the time of testing, the informant’s report is considered a valid response as long as they have witnessed the expected behavior from the child. The INAIPI team created a kit to support the assessment tool (which includes props, pictures, and everyday objects). Materials are easily found in the Dominican Republic as a strategy for upscaling data collection.

Each subtest comprises 33 items, except for language development, which has 34 items; all are arranged in ascending difficulty levels. The initial presentation of the first item is age-dependent. Once the first item on each dimension is completed, the instrument presents items in reverse until the participant successfully completes three items. Subsequently, the tool proceeds forward until the participant misses three items. This approach enables evaluators to ascertain the child’s current developmental state, identifying potential delays (backward presentation) or advanced development (forward presentation). Items must be completed in sequence as they are organized based on increasing difficulty and follow an algorithm that assesses children’s development from minimum to maximum levels. Evaluators can easily switch between different developmental areas by clicking on tabs with corresponding names.

The study’s primary aim was to determine the age range at which each item is accomplished, utilizing the Malawi MDAT norms for the initial trial. Subsequent data collection will adopt the sequence based on the findings from the present validation study.

The evaluation is conducted using a mobile device with the SIMEDID app that connects to the INAIPI server and instructs the evaluator to assess a specific child. The app calculates the first item in each development area and presents items backward and forward. Passed items were scored 1, while missed items scored 0. Items not shown (for not corresponding to the participant’s age range) were automatically completed: items before the three first achieved items were scored 1 (since it is assumed that the child has already passed these), and items after three consecutive misses scored 0 since the child is not ready to perform these.

The variables included in this study are listed in Table 1.

**Table 1.** Study variables.

Variable	Description
Age at evaluation	Numerical variable. Age is calculated in days for the analyses but displayed in months and years in the figures.
Age group	Ordinal variable. Age group was calculated by merging age in days intervals into the following month interval: 0-2, 2-4, 4-6, 6-9, 9-12, 12-15, 15-18, 18-24, 24-30, 30-36, 36-42, 42-48, 48-54, 54-60.
Service	Categorical variable. CAFI vs. CAIPI
Item success (for each item)	Categorical variable. 0 = no success, 1 = success.
Gross motor development score	Numerical variable. Scores range from 0-33, calculated by the sum of item success from the gross motor development sub-scale.
Fine motor development score	Numerical variable. Scores range from 0-33, calculated by the sum of item success from the fine motor development sub-scale.
Language development score	Numerical variable. Scores range from 0-34, calculated by the sum of item success from the language development sub-scale.
Socioemotional development score	Numerical variable. Scores range from 0-33, calculated by the sum of item success from the socioemotional development sub-scale.

No other demographic factors were considered for this analysis.

### Procedure

A total of 40 evaluators received a six-hour training session and conducted two practice evaluations. The training session comprised both theoretical and practical components. The training, facilitated by experts in training and child development, covered essential topics such as the concept of child development and SIMEDID. It included virtual follow-ups by the Child Development Evaluation Division and certification for facilitators. The module provided insights into SIMEDID’s structure, dimensions, and application in INAIPI’s service modalities. Evaluators learned about assessment processes in the children’s homes, including guidance for parents, system periodicity, and tool specifications. The practical section involved the hands-on use of evaluation kit materials in simulated assessments, emphasizing adherence to standardized data collection. The training concluded with a presentation on the SIMEDID app’s functionality, utilizing visual aids and videos to reinforce the covered content. At the end of the training, evaluators were certified as official SIMEDID evaluators.

The team conducted the recruitment through an institutional message indicating that either a CAIPI or CAFI was selected to participate. Children from CAIPI attending services during the data collection day were evaluated after a parent signed the consent form when dropping off their children at the centers. For CAFI participants, evaluation was conducted at home, and the assigned in-field INAIPI personnel contacted their families. Parents signed the informed consent before the interview took place at home. Each evaluation had a duration of 25-30 minutes.

To guarantee uniformity in assessments among evaluators, we conducted an analysis of variance (ANOVA), comparing the mean scores of participants within each age group per evaluator. The results revealed no significant differences between evaluators within each age group across all dimensions.

### Statistical methods

To determine the instrument’s psychometric properties, which correspond to the first aim, we calculated Cronbach’s alpha and split half-parallel reliability. Then, for additional evidence of content validity, we conducted descriptive analyses (means and standard deviations) of each sub-scale score for each age group to confirm the alignment of the instrument with development by age.

To determine the most appropriate item presentation order according to these data (second aim) and age-standardized norms (third aim), we conducted a logistic regression analysis on each item with item success (0 and 1) as the dependent variable and age in days as the independent variable. Following the methodology used by Gladstone *et al.* (2008, 2010), after ensuring a good model fit, the alpha and beta coefficients were used to calculate the cut-off age associated with a.9 probability of success following the formula in Equation 1:

$$P\left(Y\right)=\frac{1}{1+e^{-\left(\propto +\mathrm{\beta x}\right)}}$$ (1)

The formula was also used to calculate the .75, .50, and .25 probabilities of success for each item. We used predictive probabilities from the regressions to calculate ages corresponding to 25%, 50%, 75%, and 90% of children passing each item, as proposed by Gladstone *et al.* (2008, 2010). For the statistical analyses, we used the IBM SPSS Statistics 25 program ( https://www.ibm.com/products/spss-statistics). An open-source alternative to SPSS that can conduct the same processes is JASP ( https://jasp-stats.org/).

## Results

### Psychometric properties of SIMEDID (first aim)

To confirm the content validity of SIMEDID, we obtained descriptive statistics on each age group for the evaluated developmental areas. Table 2 shows that mean growth is progressive through age groups.

**Table 2.** Descriptive statistics for total scores by development area and age group.

Age group	n		Gross motor	Fine motor	Language	Social-emotional
0-2 month	2	Mean	*	*	*	*
		SD	*	*	*	*
2-4 month	30	Mean	**4.97**	**5.13**	**5.53**	**7.23**
		SD	2.62	3.75	1.36	3.73
4-6 month	43	Mean	**6.49**	**8.98**	**6.84**	**9.74**
		SD	2.10	2.20	1.29	3.07
6-9 month	65	Mean	**9.31**	**11.66**	**8.31**	**13.88**
		SD	2.66	2.94	2.08	4.24
9-12 month	52	Mean	**12.35**	**13.00**	**9.52**	**16.23**
		SD	1.61	1.61	1.41	2.58
12-15 month	48	Mean	**15.94**	**14.54**	**11.23**	**18.90**
		SD	4.25	2.82	2.89	3.45
15-18 month	37	Mean	**19.16**	**18.03**	**14.19**	**21.86**
		SD	4.36	5.16	5.33	3.96
18-24 month	82	Mean	**22.44**	**21.27**	**17.23**	**22.95**
		SD	3.90	4.49	6.70	4.30
24-30 month	85	Mean	**25.74**	**24.42**	**21.61**	**25.96**
		SD	4.50	4.56	6.57	4.36
30-36 month	89	Mean	**27.61**	**25.79**	**25.90**	**27.78**
		SD	3.98	3.75	5.56	3.99
36-42 month	111	Mean	**28.86**	**28.38**	**28.86**	**30.50**
		SD	3.75	3.11	4.98	3.26
42-48 month	101	Mean	**28.98**	**28.76**	**29.85**	**31.33**
		SD	4.29	3.44	5.34	2.50
48-54 month	117	Mean	**31.67**	**30.89**	**32.05**	**32.05**
		SD	3.27	3.06	3.59	2.98
54-60 month	86	Mean	**32.20**	**32.14**	**33.30**	**32.22**
		SD	1.83	1.16	1.42	2.12

* Sample size for this group was insufficient for an accurate representation.

Two internal consistency indices were calculated for each developmental area to confirm the instrument reliability, Cronbach’s alpha, and split-half Spearman-Brown’s correlation (see Table 3).

**Table 3.** Reliability of the instrument.

Development area	Cronbach's α	Split-half correlation
Gross motor	0.97	0.78
Fine motor	0.96	0.79
Language development	0.97	0.88
Socioemotional development	0.96	0.73

### Adjustment of items order presentation (second aim) and age references per item (third aim)

To complete the second and third aims, we conducted a logistic regression analysis on each item with independent variable age and dependent variable item success. The results show a good fit ( *p* < .05), except for the first item in the gross motor area and three of the first four items in the language development area.

**Figure 1.**
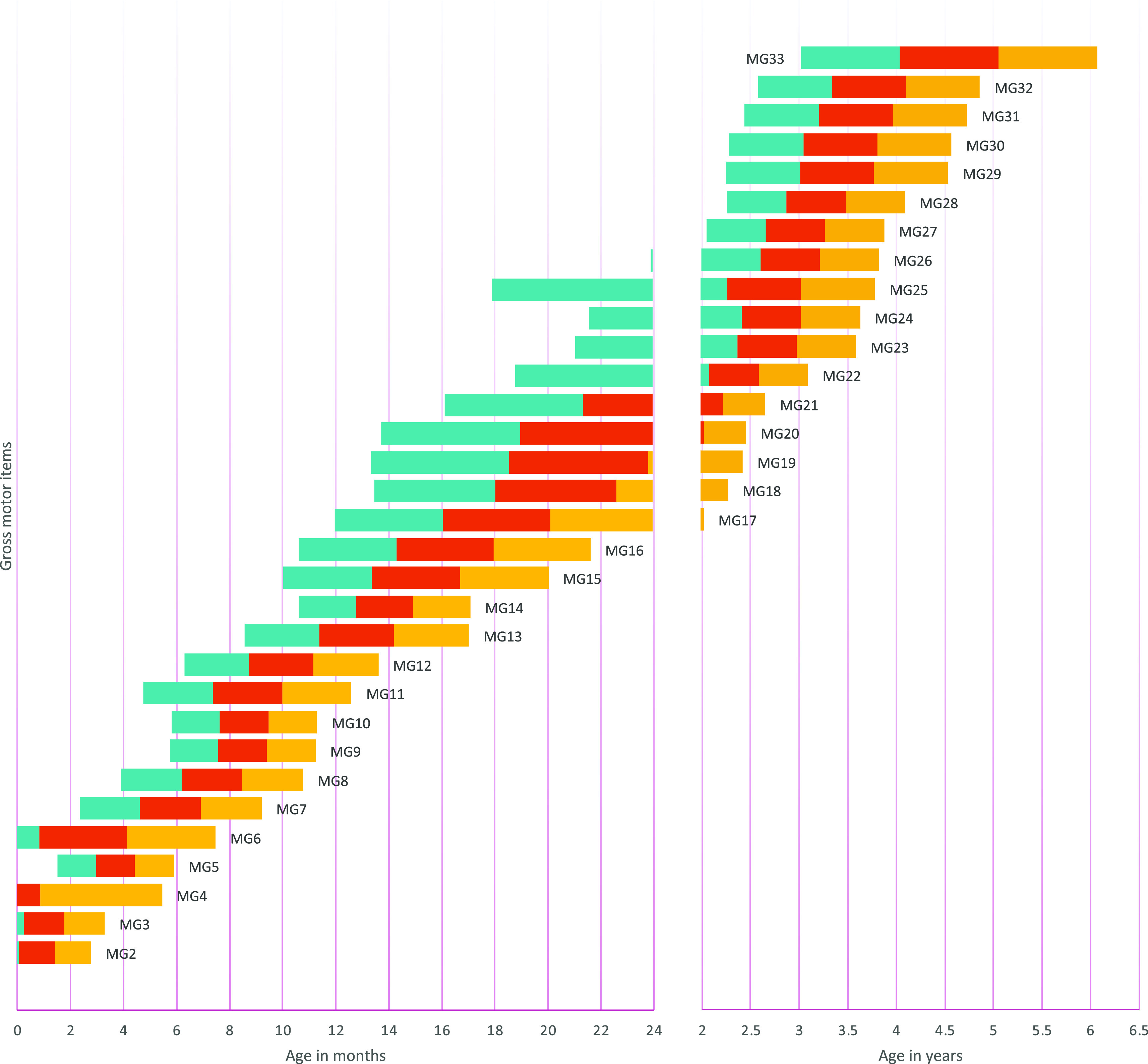
Age reference values for gross motor items. ***Notes:* MG1** Hold their head when carried; **MG2** Lift their chin off the floor; **MG3** From the prone position, they can lift their head to 90 degrees; **MG4** Support head when lifted by hands; **MG5** Flip over; **MG6** Raise head, shoulders, and chest from a prone position; **MG7** Start creeping; **MG8** Start crawling position; **MG9** Stand up with support; **MG10** Sits up unassisted; **MG11** Crawl with displacement alternating knees and hands; **MG12** Take steps with help; **MG13** Stand up unassisted; **MG14** Walk without help; **MG15** They crouch and stand up; **MG16** Walk well with cross-scroll; **MG17** Run, they may fall; **MG18** Throw ball; **MG19** Kick the ball; **MG20** Run showing coordination in their movements; **MG21** Run well, stops and start again without falling; **MG22** Jump with feet together; **MG23** Jump moving with both feet; **MG24** Stand on one foot for 3 seconds; **MG25** Stand on tiptoe with both feet; **MG26** Walk on tiptoe; **MG27** Walk in a straight line keeping balance; **MG28** Jump on one foot without support; **MG29** Can catch a ball with both hands; **MG30** Bounce and catch the ball; **MG31** Stand on one foot for 5 seconds; **MG32** Jump moving with one foot; **MG33** Jump alternating feet. The turquoise range signifies a probability of success between .25 and .50, the red range indicates a probability of success between .50 and .75, and the yellow range denotes a probability of success between .75 and .90. The y axis represents the distribution of the probability of success as explained by the color ranges.

**Figure 2.**
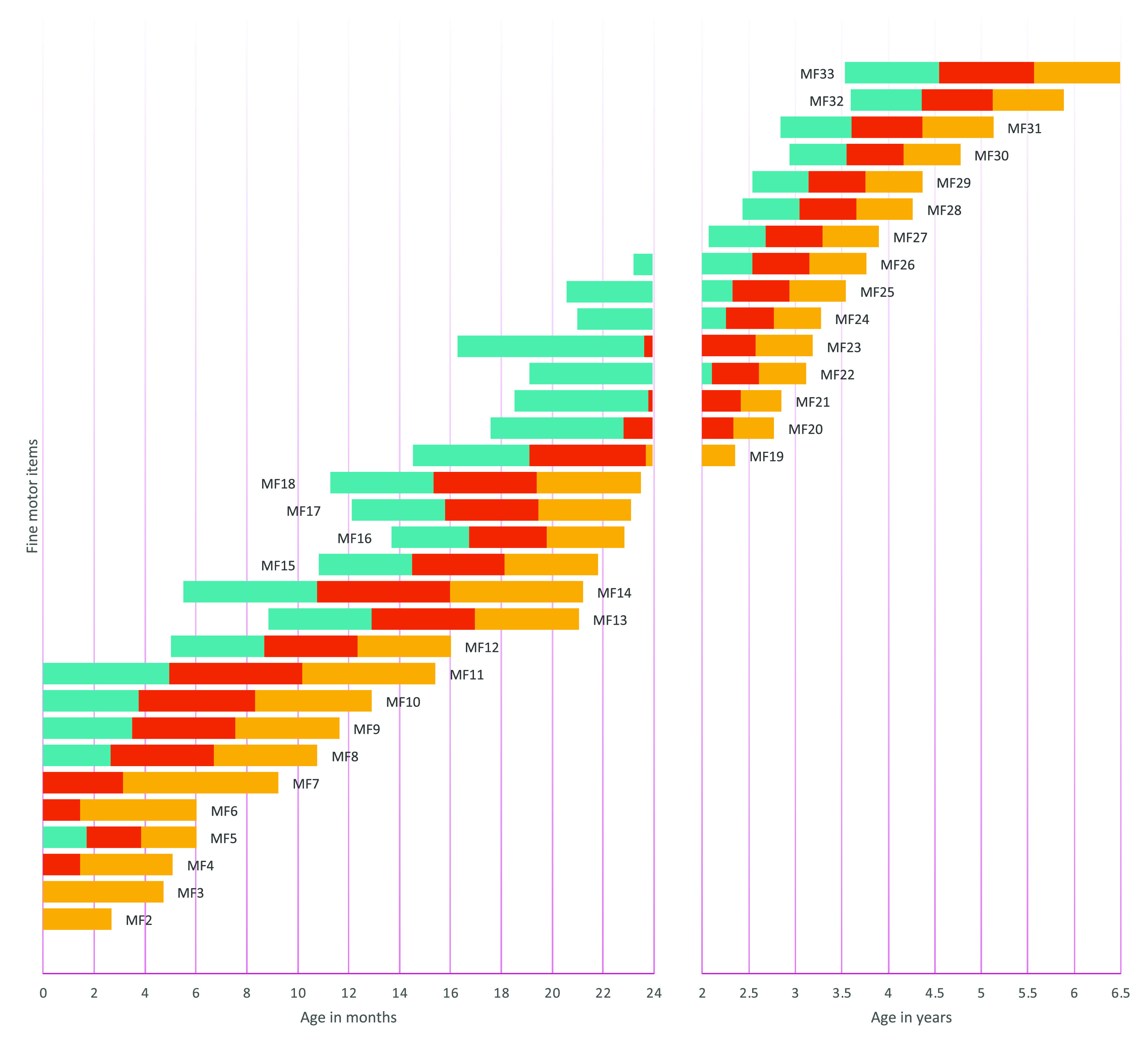
Age reference values for fine motor items. ***Notes:* MF1** Palmar grasp reflex; **MF2** Stare the midline; **MF3** Visually focuses on an object and tracks it horizontally; **MF4** Keep hands open when awake; **MF5** Hold an object in hand; **MF6** Show interest in putting an object in their mouth; **MF7** Visually focus on an object and follow it from top to bottom; **MF8** Grasp large objects voluntarily; **MF9** Hold an object in each hand; **MF10** Pass an object from one hand to another; **MF11** Pick up small objects as if their fingers were a rake; **MF12** Find the object under a blanket; **MF13** Put and take out objects from the container; **MF14** Grasp with thumb and forefinger (tweezers); **MF15** Pick up a spoon and brings it to their mouth; **MF16** Scribble; **MF17** Push a car; **MF18** Turn pages of a book; **MF19** Make a tower of two cubes; **MF20** Put nails on a board; **MF21** Make a tower of six cubes; **MF22** Make a ball of paper; **MF23** Tear paper with both hands; **MF24** Make shapes with putty; **MF25** Rotate hand to unscrew; **MF26** String; **MF27** Copy a horizontal and vertical line; **MF28** Copy a circle; **MF29** Copy a cross; **MF30** Know how to button and unbutton; **MF31** Color without leaving the outline of the drawing; **MF32** Draw a human figure; **MF33** Cut paper with scissors. The turquoise range signifies a probability of success between .25 and .50, the red range indicates a probability of success between .50 and .75, and the yellow range denotes a probability of success between .75 and .90. The y axis represents the distribution of the probability of success as explained by the color ranges.

**Figure 3.**
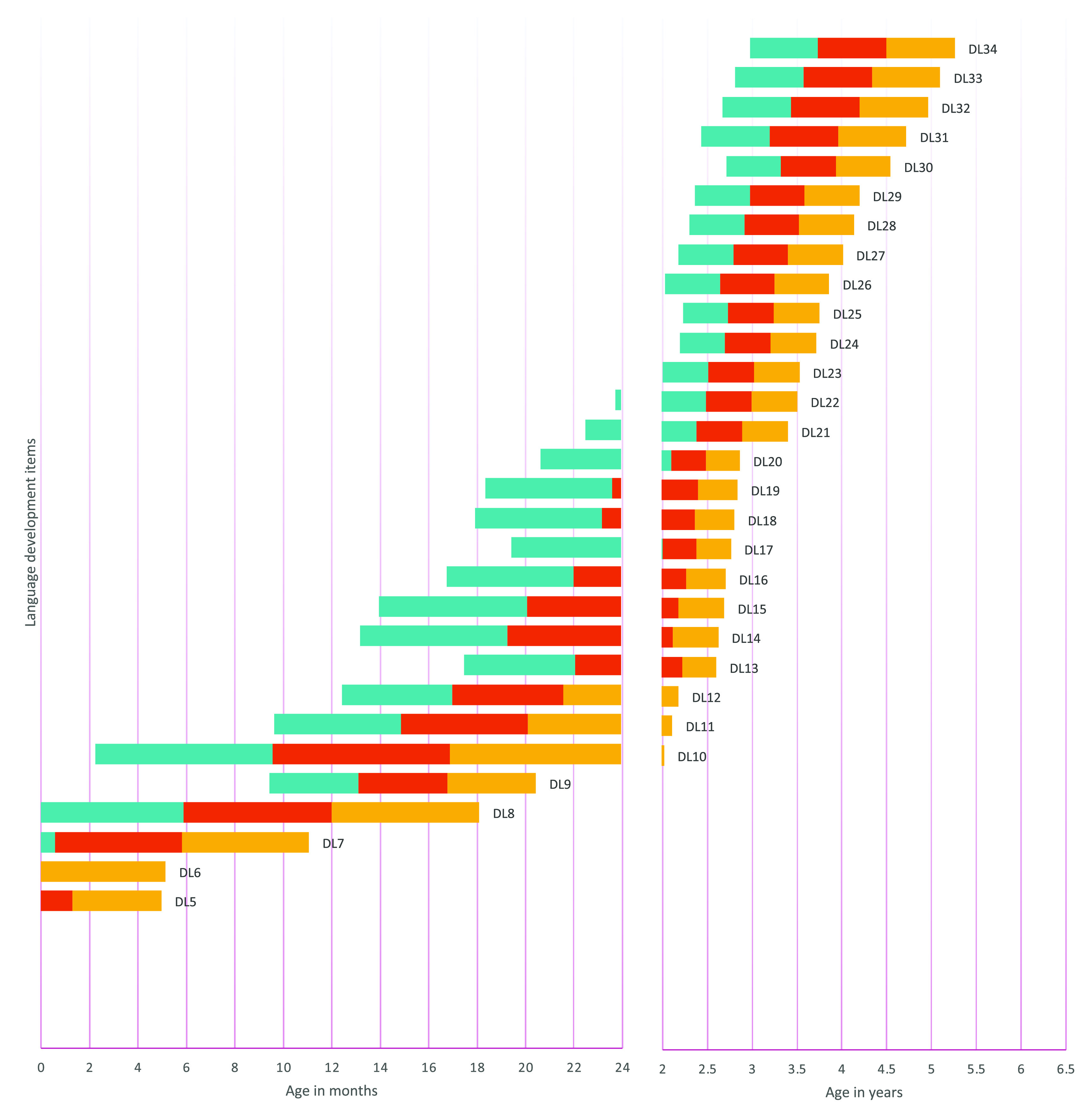
Age reference values for language development items. ***Notes:* DL1** Calm down when speaking to them; **DL2** Startle or jump in response to sounds; **DL3** Cry to express needs; **DL4** They laugh; **DL5** Make sounds with the throat; **DL6** Turn their head when they search for a sound; **DL7** React when called by their name; **DL8** Pronounce syllables like Ma, Pa, Ba, Ta; **DL9** Point with their finger when they want something; **DL10** Repeat the same syllable twice “Dada, Mama, Mimi, Tata, Papa, Yaya, Baba”; **DL11** Understand the meaning of the word no; **DL12** Follow one-step commands; **DL13** Follow two-step instructions; **DL14** Answer with yes or no; **DL15** Pronounce their first words with communicative intention; **DL16** Recognize at least 6 objects; **DL17** Point to 5 parts of their body; **DL18** Use a two-word phrase; **DL19** Say 6 words; **DL20** Say their name; **DL21** Know the use of three or more objects; **DL22** Can identify 10 objects by name; **DL23** Pronounce sentences of three words; **DL24** Use more than 15 words; **DL25** Use long sentences; **DL26** Know the qualities or characteristics of an object; **DL27** Pronounce the sounds of words correctly; **DL28** Describe the drawing; **DL29** Name at least three things in a category; **DL30** Recognize opposites; **DL31** Can count up to 5 or more objects; **DL32** Answer two comprehension questions; **DL33** Compare objects; **DL34** Tell a story from a sequence of images. The turquoise range signifies a probability of success between .25 and .50, the red range indicates a probability of success between .50 and .75, and the yellow range denotes a probability of success between .75 and .90. The y axis represents the distribution of the probability of success as explained by the color ranges.

**Figure 4.**
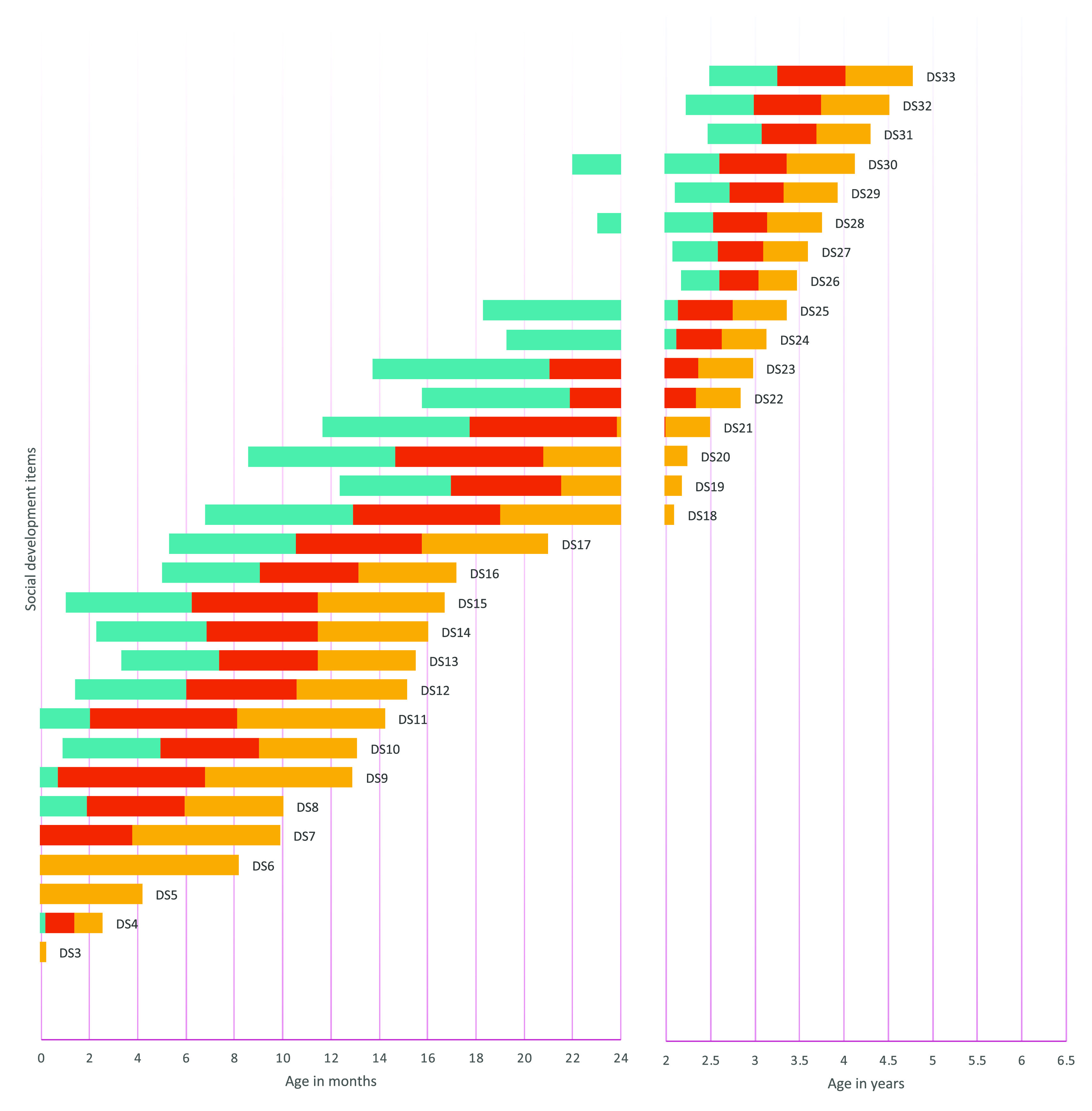
Age reference values for socioemotional development items. ***Notes:* DS1** Calm down with family members or caregivers; **DS2** Smile spontaneously; **DS3** Smile in response to a person; **DS4** Recognize the voice of the main caregiver; **DS5** Make eye contact; **DS6** Touch the examiner's hands; **DS7** They are aware of their hands (body); **DS8** Try to hold a cup when being fed; **DS9** Respond to a conversation; **DS10** Raise their arms or indicate that they want to be carried; **DS11** Laugh out loud; **DS12** Explore their face when they are in front of the mirror; **DS13** Show interest or intention to feed themselves; **DS14** Look for continuing the game; **DS15** Explore the environment; **DS16** Participate in games; **DS17** Wave or verbally greet; **DS18** Express their satisfaction when they achieve something; **DS19** Take a glass without spilling; **DS20** Imitate adult actions; **DS21** Recognize their belongings; **DS22** Express interest in playing with other children; **DS23** Symbolic game; **DS24** Refer to themselves as “I”; **DS25** Say the names of the people with whom they live; **DS26** They urinate or defecate independently without dirtying their clothes; **DS27** Indicate in some way that they need to urinate or defecate; **DS28** Identify basic emotions in images; **DS29** Come up with games; **DS30** Share their belongings; **DS31** Recognize basic emotions in themselves and express them verbally; **DS32** Recognize and express basic emotions in others; **DS33** Participate in games respecting rules and turns. The turquoise range signifies a probability of success between .25 and .50, the red range indicates a probability of success between .50 and .75, and the yellow range denotes a probability of success between .75 and .90. The y axis represents the distribution of the probability of success as explained by the color ranges.

The item presentation order was determined by sorting the age at which each item had a .9 probability of success from the results of logistic regression analyses on each item. To describe the expected evolution of item success probability across age, we also determined the .75, .50, and .25 probability of success. We thus provided the range amplitude for each item’s success predicted by age. Figures 1 to 4 contain a visual representation of sorted items and corresponding probabilities of success. Note that results are presented in two scales for the X axis: for the first two years, the X axis corresponds to age in months, and for the following age ranges, in years.

## Discussion

This paper collects evidence for the validity of SIMEDID, an electronic early childhood development screening tool adapted to the Dominican context conducted by INAIPI’s personnel through an electronic application. Regarding the first aim, we found that the instrument has adequate psychometric properties: the instrument’s subscales showed high internal consistency scores, evidencing excellent reliability. Furthermore, total scores for each sub-scale increased progressively across age, which evidenced alignment with standards already provided by an expert panel ( Alonso *et al.,* 2022) and criterion validity with a previous version of this instrument on a small sample size ( Sánchez-Vincitore *et al.,* 2019).

Regarding the second aim, we found that age predicted item success in most items, which supports similar results in children from Malawi Gladstone *et al.* (2008, 2010). However, age did not predict four initial items from the gross motor and language development subscales. We attribute these null findings to the fact that our study did not include an acceptable sample size for the age group for which these items were relevant. This is because INAIPI’s services to very young children were scarce at the time of this study, which will be considered for future studies. To comply with the third aim, age-standardized norms for each item were established, obtaining correspondences between ages and different probabilities of success for each item, which will allow comparing the achievement of participants with what is expected at their age.

Adapting this instrument to the Dominican context guarantees that cultural aspects of childrearing do not overshadow developmental scores ( Suchdev *et al.,* 2017) and that development is not under or overestimated. Having the instrument in an electronic platform solves two main challenges. First, personnel training is kept to the minimum since the platform guides the evaluator throughout the evaluation, presenting the items that only pertain to the child according to their age, with suggested videos and additional testing resources. Second, having SIMEDID connected to INAIPI servers and incorporating the data on national services provided by INAIPI creates a continuous stream that otherwise would be costly and logistically convolute data. This data stream will allow the development of new research agendas that include correlational modeling, intervention studies, and longitudinal studies to understand better the factors associated with childhood development in the Dominican Republic in a timely and cost-efficient way.

The study findings demonstrate that SIMEDID passes three elements of a checklist of critical methodological elements to consider when appraising a childhood development assessment tool: (1) the instrument measures domains affected by the risk factor or intervention; (2) reliability and validity of the instrument in the population of interest; (3) sensitivity of the instrument to identify changes; (4) logistics and methodology is suitable for evaluating the outcome; and (5) consideration of control group ( Sabanathan *et al.,* 2015). SIMEDID passes the first two elements from this checklist, as it measures specific domains of early childhood development previously identified as risk factors in the Dominican Republic, such as the sociodemographic and psychosocial factors that predict childhood development ( Sánchez-Vincitore and Castro, 2022) and low levels of oral language comprehension in school-aged children that should be addressed during early childhood development before children enter primary school ( Sánchez-Vincitore *et al.,* 2020, 2022) among other risk factors. In addition, it passes the fourth element, given that the logistic and methodology was specifically designed to assess the outcome in the Dominican context. Future studies will address the third and fifth items when SIMEDID is used as a monitoring tool on a population basis.

This study has some limitations that should be considered before its interpretation. Children from the sample for which these age standards were obtained received services at INAIPI. Socio-economic vulnerability is one of the main criteria for receiving such services. This means that the sample may not accurately reflect all children in the Dominican Republic since it comprises only those involved in INAIPI programs within the metropolitan region. During the study period, 77,000 children enrolled in INAIPI from the metropolitan region accounted for 1.23 percent of the total population of children ages 0 to 5. The limited sample size was influenced by the challenges posed by the COVID-19 pandemic, including restrictions and prevention measures. Future studies should consider the whole socio-economic position spectrum to obtain national norms. The data generated in the Dominican context using SIMEDID has limited comparability to data from other countries.

Another important limitation is that the experience of creating this platform in the Dominican Republic was cost-effective due to the already existing infrastructure within INAIPI, which should be considered when transferring it to other countries. The institution is the national administrator of early childhood services, which gives them access to the population of interest and trained personnel already working with children. In addition, INAIPI has the Division of Early Childhood Development Measurement, with dedicated personnel to designing, creating, supervising, and training the personnel in childhood development measurement. Also, INAIPI has a dedicated Information and Communications Technology Department, which developed the online platform and made it synchronized with SIGEPI, the national database for managing data from early childhood services. Further studies should conduct a cost-per-user analysis to evaluate its efficiency.

Finally, the study did not account for other relevant factors in childhood development, such as low birth weight and other health factors or disabilities. However, future data collection activities, which will involve a larger sample of children from INAIPI, will consider variables such as birth weight, prematurity, and other health factors to determine cutoff points that better identify children at risk for developmental delays and to further understand the sensitivity of SIMEDID.

Additionally, we could not validate the initial items of SIMEDID given that INAIPI does not provide services to infants younger than 45 days, given that mothers are on maternity leave during that period. However, the research team is meeting other governmental institutions that work with infants that young to expand the age range of the study.

We have yet to demonstrate the tool’s ability to sensitively identify changes, as this is the first large-scale data collection using this instrument. However, since SIMEDID is already part of the monitoring strategy, with constant data collection, it would be relatively simple to demonstrate its sensitivity to changes.

Even with these limitations, these results will allow the pertinent institutions of the Dominican Republic to implement and report more accurate early childhood development indicators. They will also contribute to creating a robust monitoring system with a high-quality data collection process that allows evidence-based and timely decision-making. Furthermore, such a system will contribute to generating longitudinal data that can establish the association between childhood development and sociodemographic and psychosocial variables and determine the impact of initiatives and interventions ( Richter *et al.,* 2017), which is not sufficiently evaluated in most countries ( Daelmans *et al.,* 2017).

In the future, SIMEDID will identify children at risk for developmental delays using data from a standardization study which will incorporate the item presentation order determined by the current validation study. Ensuring the appropriate order will narrow standard deviations, enabling the determination of developmental cut-off points at -1 and -2 standard deviations for different levels of developmental delay risk.

Although SIMEDID was created to be integrated into the services provided by INAIPI, and the instrument so far has only been administered to INAIPI beneficiaries, efforts to make a paper version of SIMEDID are on the way under the name of TADID (Tamizaje de Desarrollo Infantil Dominicano). This will allow other institutions, clinicians, schools, and pediatricians to use this validated tool at no cost.

## Conclusion

This study provides compelling evidence for the validity and utility of SIMEDID, an electronic early childhood development screening tool adapted to the Dominican context. Demonstrating robust psychometric properties and high internal consistency, SIMEDID aligns with expert panel standards and exhibits criterion validity. Age-standardized norms enhance its precision, allowing nuanced assessments of developmental progress. The cultural adaptation addresses potential biases in childrearing practices. Future considerations include broader accessibility through the TADID paper version and the tool’s potential contribution to generating longitudinal data for evidence-based decision-making. Overall, SIMEDID emerges as a promising and versatile screening tool with implications for both program design and individual monitoring of childhood development in the Dominican Republic.

In conclusion, this study provides evidence for the validity of SIMEDID, an electronic early childhood development screening tool adapted to the Dominican context, with adequate psychometric properties and age-standardized norms for each item. Adapting this instrument to the Dominican context ensures that cultural aspects of childrearing do not overshadow developmental scores. While SIMEDID is a screening tool and not intended for diagnosis, it offers valuable insights for caregivers and stakeholders, both at a group level for program design and decision-making, as well as at the individual level to monitor each child’s progress.

## Data Availability

Open Science Framework: Database for Validation of the Dominican System for Measuring Early Childhood Development. https://doi.org/10.17605/OSF.IO/KW3B8 ( Sánchez-Vincitore *et al., *2023b). The project contains the following underlying data:
• Codebook SIMEDID.docx (names and values of each variable). • Database – Validation study – SIMEDID.csv (database). Data are available under the terms of the Creative Commons Attribution 4.0 International license (CC-BY 4.0). Open Science Framework: Extended data - Validation of the Dominican system for measuring early childhood development. https://doi.org/10.17605/OSF.IO/SWN8C ( Sánchez-Vincitore *et al., *2023a). This project contains the following extended data:
• SIMEDID – Presentation order V2.xlsx. (Order of item presentation before and after data collection. Spanish and English translations) • Extended data - COSMIN checklist.pdf Data are available under the terms of the Creative Commons Attribution 4.0 International license (CC-BY 4.0).

## References

[ref1] Alonso MA Valdez ME Jiménez A : Creación del Sistema de Medición de Desarrollo Infantil Dominicano (SIMEDID). *Ciencia y Educación.* 2022;6(3):71–77. 10.22206/CYED.2022.V6I3.PP71-77

[ref2] Daelmans B Darmstadt GL Lombardi J : Early childhood development: The foundation of sustainable development. *Lancet.* 2017;389(10064):9–11. 10.1016/S0140-6736(16)31659-2 27717607

[ref3] Inter-American Dialogue: *Medición del desarrollo infantil en América: Desafíos para la medición y respuestas de política.* Washington DC: Inter-American Dialogue;2020.

[ref4] Gladstone M Lancaster GA Jones AP : Can Western developmental screening tools be modified for use in a rural Malawian setting? *Arch. Dis. Child.* 2008;93(1):23–29. 10.1136/adc.2006.095471 17379661

[ref5] Gladstone M Lancaster GA Umar E : The Malawi developmental assessment tool (MDAT): The creation, validation, and reliability of a tool to assess child development in rural African settings. *PLoS Med.* 2010;7(5):e1000273. 10.1371/journal.pmed.1000273 20520849 PMC2876049

[ref24] INAIPI : Informe Piloto de Medición del desarrollo infantil. 2020. [Unpublished manuscript].

[ref6] Loizillon A Petrowski N Britto P : Development of the Early Childhood Development Index in MICS surveys. MICS Methodological Papers, No. 6. 2017.

[ref7] Lokuketagoda BUWP Thalagala N Fonseka P : Early development standards for children aged 2 to 12 months in a low-income setting. *SAGE Open.* 2016;6(4):215824401667312. 10.1177/2158244016673128

[ref8] Molina Achécar M Polanco JJ : Encuesta por Conglomerados de Indicadores Múltiples (MICS-2000). 2001.

[ref9] ONE, & UNICEF: Encuesta nacional de hogares de propósitos múltiples - Encuesta de Indicadores Múltiples por Conglomerados 2014, Informe final. 2016.

[ref10] ONE, & UNICEF: ENHOGAR-MICS Encuesta de Indicadores Múltiples por Conglomerados 2019, Informe de resultados de la encuesta. 2021.

[ref11] Richter LM Daelmans B Lombardi J : Investing in the foundation of sustainable development: Pathways to scale up for early childhood development. *Lancet.* Elsevier B.V;2017;389(10064):103–118. 10.1016/S0140-6736(16)31698-1 27717610 PMC5880532

[ref12] Sabanathan S Wills B Gladstone M : Child development assessment tools in low-income and middle-income countries: How can we use them more appropriately? *Arch. Dis. Child.* 2015;100(5):482–488. 10.1136/archdischild-2014-308114 25825411 PMC4413834

[ref25] Sánchez-Vincitore LV : Propiedades psicométricas del tamizaje de desarrollo infantil del Sistema de Medición de Desarrollo Infantil Dominicano (SIMEDID). 2020. [Unpublished manuscript].

[ref13] Sánchez-Vincitore LV Castro A : The role of sociodemographic and psychosocial variables in early childhood development: A secondary data analysis of the 2014 and 2019 Multiple Indicator Cluster Surveys in the Dominican Republic. *PLOS Global Public Health.* 2022;2(7):e0000465. 10.1371/journal.pgph.0000465 36962194 PMC10021185

[ref14] Sánchez-Vincitore LV Mencía-Ripley A Veras-Díaz C : Efectos de una intervención de alfabetización en las habilidades lectoras de estudiantes de primaria: Proyecto USAID Leer. *Revista Caribeña de Investigación Educativa.* 2020;4(2):78–95. 10.32541/recie.2020.v4i2.pp78-95

[ref15] Sánchez-Vincitore LV Schaettle P Castro A : Validation of the Malawi Developmental Assessment Tool for children in the Dominican Republic: Preliminary results. *PLoS One.* 2019;14(8):e0221162. 10.1371/journal.pone.0221162 31415641 PMC6695133

[ref16] Sánchez-Vincitore LV Veras C Mencía-Ripley A : Reading comprehension precursors: Evidence of the simple view of reading in a transparent orthography. *Front. Educ.* 2022;7:914414. 10.3389/feduc.2022.914414

[ref20] Sánchez-Vincitore LV Alonso Pellerano MA Valdez ME : Extended data - Validation of the Dominican system for measuring early childhood development.Dataset.2023a, February 21. 10.17605/OSF.IO/SWN8C PMC11036040 38655207

[ref21] Sánchez-Vincitore LV Alonso Pellerano MA Valdez ME : Database for Validation of the Dominican System for Measuring Early Childhood Development.Dataset.2023b, February 7. 10.17605/OSF.IO/KW3B8 PMC11036040 38655207

[ref22] Sireci SG Sukin T : Test validity. Geisinger KF Bracken BA Carlson JF , editors. *APA handbook of testing and assessment in psychology, Vol. 1. Test theory and testing and assessment in industrial and organizational psychology.* American Psychological Association;2013; pp.61–84. 10.1037/14047-004

[ref17] Suchdev PS Boivin MJ Forsyth BW : Assessment of neurodevelopment, nutrition, and inflammation from fetal life to adolescence in low-resource settings. *Pediatrics.* 2017;139:S23–S37. 10.1542/peds.2016-2828E 28562246

[ref18] UNESCO Institute of Statistics (UIS): Proportion of children aged 24-59 months who are developmentally on track in health, learning and psychosocial well-being, by sex. n.d. Reference Source

[ref19] United Nations: Transforming our world: The 2030 agenda for sustainable development (A/RES/70/1). 2015. Reference Source

