# Supplementary material for: Validation of the Dominican system for measuring early childhood development
Source: F1000Res. 2024 Jan 29;12:279. Originally published 2023 Mar 14. [Version 2] doi: 10.12688/f1000research.128657.2 (PMC11036040; doi:10.12688/f1000research.128657.2)
Supplement: Peer review history

# Reviewer report: Reviewer response for version 2

**DOI:** 10.5256/f1000research.161800.r241966

**Published:** 2024-04-22

**Authors:** Melissa J. Gladstone

**Affiliations:** 1 Department of Women and Children's Health, Institute of Life Course and Medical Sciences, University of Liverpool, Liverpool, UK

Laura Sanchez-Vincitore and team have worked hard to address comments and the paper is now much better than it was and clearer for the reader to understand the work done. I agree that the results need to consider the epidemiology of the population utilised for this study and that future studies in other populations or larger more well characterised populations may help with this.

Is the work clearly and accurately presented and does it cite the current literature?

Partly

If applicable, is the statistical analysis and its interpretation appropriate?

Partly

Are all the source data underlying the results available to ensure full reproducibility?

No

Is the study design appropriate and is the work technically sound?

Partly

Are the conclusions drawn adequately supported by the results?

No

Are sufficient details of methods and analysis provided to allow replication by others?

No

Reviewer Expertise:

Neurodevelopmental Paediatrics and International Child Health

I confirm that I have read this submission and believe that I have an appropriate level of expertise to confirm that it is of an acceptable scientific standard.

# Reviewer report: Reviewer response for version 2

**DOI:** 10.5256/f1000research.161800.r241965

**Published:** 2024-03-13

**Authors:** Mphelekedzeni Caroline Mulaudzi

**Affiliations:** 1 Paediatrics and Child Health, Charlotte Maxeke Johannesburg Academic Hospital, Johannesburg, Gauteng, South Africa; 2 University of Witwater'srand, Johannesburg, South Africa

I commend the researchers for addressing my previous concerned very well.

 I am still of the opinion that

 1. the age standardization the way it was done may underestimate the developmental potential because known physical and medical risk factors were not excluded in the sample for age standardization 

 2. the SIMEDID is adequate and sufficient for the domains tested. older children around 5 year may need more on cognitive.

 In conclusion the above points are for the researchers to consider in their future projects as stated in the article.

Is the work clearly and accurately presented and does it cite the current literature?

Partly

If applicable, is the statistical analysis and its interpretation appropriate?

Partly

Are all the source data underlying the results available to ensure full reproducibility?

Partly

Is the study design appropriate and is the work technically sound?

Partly

Are the conclusions drawn adequately supported by the results?

Partly

Are sufficient details of methods and analysis provided to allow replication by others?

Partly

Reviewer Expertise:

Child health and Early Childhood Development

I confirm that I have read this submission and believe that I have an appropriate level of expertise to confirm that it is of an acceptable scientific standard.

# Reviewer report: Reviewer response for version 1

**DOI:** 10.5256/f1000research.141269.r194708

**Published:** 2023-08-31

**Authors:** Mphelekedzeni Caroline Mulaudzi

**Affiliations:** 1 Paediatrics and Child Health, Charlotte Maxeke Johannesburg Academic Hospital, Johannesburg, Gauteng, South Africa; 2 University of Witwater'srand, Johannesburg, South Africa

I commend the researchers for doing research in the area of instruments for early child development in low/middle-income countries.

**Literature review**

 The researchers should have shared more on their initial work of adapting MDAT to the Dominican instrument and how it differs.

**Methodology and statistical analysis**

 There is no description of the sampling technique used and no information on how they determined the population was normal

 As far as whether the correct statistical analysis was done to meet the aims of the study.

 There is clear information and a report on reliability but not validity.

**Results**

 Figures should be labeled appropriately with an explanation of the range of colors' meanings.

 The Y axis of the item should not only mention the domain but also what analysis

**Discussion**

 Interpretation of the result is poor. The researchers gave results of the Chronbach alpha and split-half correlation but did not elaborate on the relevance

 They did not comment on how the instrument should be used in the future, especially with items that had a standard deviation that was higher than 3 and even some items that had a standard deviation greater or equal to 6.

Is the work clearly and accurately presented and does it cite the current literature?

Partly

If applicable, is the statistical analysis and its interpretation appropriate?

Partly

Are all the source data underlying the results available to ensure full reproducibility?

Partly

Is the study design appropriate and is the work technically sound?

Partly

Are the conclusions drawn adequately supported by the results?

Partly

Are sufficient details of methods and analysis provided to allow replication by others?

Partly

Reviewer Expertise:

Child health and Early Childhood Development

I confirm that I have read this submission and believe that I have an appropriate level of expertise to confirm that it is of an acceptable scientific standard, however I have significant reservations, as outlined above.

# Response

**Published:** 2024-01-23

**Authors:** Laura V. Sánchez-Vincitore

**Affiliations:** Universidad Iberoamericana, Dominican Republic

Dear Dr. Mulaudzi, 

 We thank you for your comments and questions, which have helped us improve the quality of our manuscript. We address your questions below and in the manuscript:

**1. The researchers should have shared more on their initial work of adapting MDAT to the Dominican instrument and how it differs. ** 

 How was this addressed in the manuscript?

 We have described the process of adapting the instrument in more detail, summarizing Alonso et al. 2022.  

**2. There is no description of the sampling technique used and no information on how they determined the population was normal**

 How was this addressed in the manuscript?

 We used an intentional sampling with an N=948. We have clarified that the sample came from INAIPI beneficiaries who lived in the capital city of Santo Domingo and provided additional details.  

**3. There is clear information and a report on reliability but not validity. ** 

 How was this addressed in the manuscript?

 We have included additional details on the creation of SIMEDID that includes more validity information, such as collaboration with experts, content analysis, and ongoing data collection activities. As SIMEDID transitions into the national surveillance system, continued data collection will contribute further evidence of its validity. 

**4. Figures should be labeled appropriately with an explanation of the range of colors' meanings. ** 

 How was this addressed in the manuscript?

 We have added a note to each figure explaining the color range.

**5. The Y axis of the item should not only mention the domain but also what analysis**

 How was this addressed in the manuscript?

 The Y represents the distribution of the probability of success, as explained by the color ranges. We have added this information to each figure. 

****

**6. Interpretation of the result is poor. The researchers gave results of the Chronbach alpha and split-half correlation but did not elaborate on the relevance**

 How was this addressed in the manuscript?

 We have expanded the conclusion with an additional interpretation of the results.  

**7. They did not comment on how the instrument should be used in the future, especially with items that had a standard deviation that was higher than 3 and even some items that had a standard deviation greater or equal to 6.**

 How was this addressed in the manuscript?

 In the future, SIMEDID will identify children at risk for developmental delays. The current study aimed to enhance item presentation order. The observed large standard deviations may result from presenting items in an order that doesn't fully adapt to the Dominican context. The upcoming standardization study, which includes a more fitting presentation order of items obtained in the present validation study, seeks to establish national norms by calculating cut-off points based on standard deviations once the presentation order is appropriately configured. We have explained these plans in the discussion.

# Reviewer report: Reviewer response for version 1

**DOI:** 10.5256/f1000research.141269.r166639

**Published:** 2023-04-11

**Authors:** Melissa J. Gladstone

**Affiliations:** 1 Department of Women and Children's Health, Institute of Life Course and Medical Sciences, University of Liverpool, Liverpool, UK

This article provides information about the recent validation of a system for measuring child development in Dominican Republic.

 This is quite a large study which provides some initial information as to how a new tool for measuring child development in the Dominican Republic is working. This is extremely laudable and very important. Tools such as this are required to ensure that better developmental surveillance can be done across countries globally. 

 I do have some reservations that could be addressed but presently the article is not in a fit state to publish. I outline these below. 

**Methods:**

 It is not clear if the tool has been adapted from the mentioned MDAT tool and whether it is parent report or directly observed or a mixture of the two. It is not clear if it needs a kit to support assessment or not. It is also not clear if there are any pictures or materials that have been created to support assessment. It is also not entirely clear if it has been piloted prior to this use.

 The authors describe how the tool was reviewed by an expert pane to provide information on construct validity but also to revise the items and their definitions. The authors provide examples of items they modified but it is not clear what the initial item was. It might be helpful to have a list of items and what was and was not changed. It also might be helpful to use the COSMIN framework to clarify the various validation procedures to ensure they were done as would be recommended and if not, this could be discussed more in the discussion part of the paper.

 Do the items have to be passed in succession? Can they move from one area to another within the app?

**Methods**

 The authors describe that a Normative sample of children were assessed. Can the authors be clearer about how this is known. It looks like the children who were assessed were those who attended Comprehensive Care Centers for Early Childhood but it is not clear how much or little these represent the general population of children in the Dominican Republic. The authors do not make it clear if any other demographics were taken into account e.g. born prematurely? Birth weight? Adversities and sociodemographic status (maternal education), health status and frequency of admissions to hospital, nutritional status etc?

 The authors describe the use of Cronbach’s alpha internal reliability and “split half parallel reliability” but it is not clear if any reliability was done at the time of training and whether any quality control was done between examiners?

 What did the 6 hour session for training consist of? And could it be clearer who did this and what is needed for the future? Were there online sessions? In person sessions? Any materials that were created? Certification of assessors?

 Furthermore, could the authors provide more information about whether children were assessed with a disability and if not, how did they decide to exclude them? Can the present tool go down further to assess a child who is not performing? How did the team decide if they had a child that they felt was below the norm?

**Discussion**

 The authors describe that age did not predict certain items. Is this because these items are not acceptable or work in the setting or is it because there were not enough children to assess these items? It seems the latter.

 The authors conclude that the study passes three elements which include that it is reliable and valid. The authors may want to check the COSMIN criteria to make decisions on what reliability and validity they are describing as having definitively concluded on through this study. It is not clear that the authors have definitively shown that the tool can sensitively identify changes? Could they make that clearer and that they have demonstrated its use in another population who are known to have a problem through doing a Gold Standard assessment of these children.

 Could the authors provide a little more information on what they plan to do with the info from this study? At the moment, it seems like it goes into a data collection system but what and how will they create “cut offs” decided for referral? These next steps are very important and could be provided more clearly for the reader.

Is the work clearly and accurately presented and does it cite the current literature?

Partly

If applicable, is the statistical analysis and its interpretation appropriate?

Partly

Are all the source data underlying the results available to ensure full reproducibility?

No

Is the study design appropriate and is the work technically sound?

Partly

Are the conclusions drawn adequately supported by the results?

No

Are sufficient details of methods and analysis provided to allow replication by others?

No

Reviewer Expertise:

Neurodevelopmental Paediatrics and International Child Health

I confirm that I have read this submission and believe that I have an appropriate level of expertise to state that I do not consider it to be of an acceptable scientific standard, for reasons outlined above.

# Response

**Published:** 2024-01-23

**Authors:** Laura V. Sánchez-Vincitore

**Affiliations:** Universidad Iberoamericana, Dominican Republic

Dear Dr. Gladstone, 

 We are thankful for your constructive feedback, which has contributed to improving our manuscript. We have addressed all your questions and comments, as explained below and included in the manuscript’s new version. 

***1. It is not clear if the tool has been adapted from the mentioned MDAT tool.***

  The SIMEDID tool has been adapted from the MDAT tool and other instruments, as reported by Alonso et al. 2022, which provides more details on the process of adaptation and validation. 

 How was this addressed in the manuscript?

 We described the process of adapting the instrument in more detail, summarizing Alonso et al. 2022. 

***2. It is not clear if the assessment tool requires direct observation or is based on parent***

***report, or a combination of both.***

 The SIMEDID tool combines direct observation of the child's behavior by educational agents and reports from individuals who have spent enough time with the child. That person must have observed the desired behavior. For example, in INAIPI’s case, the evaluator is the person in contact with the child, the educational agent, the community agent, and/or the parent.  

 How was this addressed in the manuscript?

 We included the following statement in the methods section: “The assessment tool requires an observation of the child’s behavior, either by the evaluator, parent, or the person in charge of the child at a given time. In case the child does not show the expected behavior at the time of testing, the informant’s report is considered a valid response as long as they have witnessed the expected behavior from the child.”  

***3. It is not clear if the assessment tool requires a kit to support the assessment or not.***

   

 Yes, there is a kit available to support the assessment with the SIMEDID tool. 

 How was this addressed in the manuscript?

 We included the following statement in the methods section: “The INAIPI team created a kit to support the assessment tool (which includes props, pictures, and everyday objects). Materials are easily found in the Dominican Republic as a strategy for upscaling data collection.” 

***4. It is not entirely clear if there are any pictures or materials that have been created to support the assessment.***

 Yes, the SIMEDID tool includes pictures and materials to support the assessment. 

 How was this addressed in the manuscript?

 See comment #3. 

***5. It is not entirely clear if the assessment tool has been piloted before this use.***

 The SIMEDID assessment tool underwent an initial pilot study involving 100 children before its use in this study. The pilot study, which is unpublished, provided valuable insights into the tool's performance and informed the validation process.  

 How was this addressed in the manuscript?

 The following statement has been included “The pilot study included 100 children aged 45 days to 5 years who were receiving INAIPI services. The study confirmed the platform’s offline functionality, speed, and user-friendly interface, along with the willingness of educators and animators to participate. In addition, the instrument showed good internal consistency.” 

***6. It might be helpful to have a list of items and what was and was not changed.***

   

 The extended data now include a list of the items in the SIMEDID tool and details on what was and was not changed during the adaptation process. 

 How was this addressed in the manuscript?

 The extended data are referenced in the manuscript.  

***7. It might be helpful to use the COSMIN framework to clarify the various validation procedures to ensure they were done as would be recommended.***

 A new supplementary table that follows the COSMIN framework has been included in the extended data. This table will serve as a live document to track data collection activities and ensure that the instrument continues to improve over time. 

 How was this addressed in the manuscript?

 The extended data are referenced in the manuscript.  

***8. Do the items have to be passed in succession? Can they move from one area to another within the app?***

 Items must be completed in sequence as they are organized based on increasing difficulty and follow an algorithm that assesses children's development from minimum to maximum levels. 

 Evaluators can easily switch between different developmental areas by clicking on tabs with corresponding names. However, to progress to the next item, the current one must be answered. Additionally, to finish the test and submit results, all age-specific items must be filled out. Any missing responses will trigger an alert from the platform. 

 How was this addressed in the manuscript?   

 We included the following statement in the instruments section: 

 “Each subtest comprises 33 items, except for language development, which has 34 items; all are arranged in ascending difficulty levels. The initial presentation of the first item is age-dependent. Once the first item on each dimension is completed, the instrument presents items in reverse until the participant successfully completes three items. Subsequently, the tool proceeds forward until the participant misses three items. This approach enables evaluators to ascertain the child's current developmental state, identifying potential delays (backward presentation) or advanced development (forward presentation). Items must be completed in sequence as they are organized based on increasing difficulty and follow an algorithm that assesses children's development from minimum to maximum levels. Evaluators can easily switch between different developmental areas by clicking on tabs with corresponding names.  

 The study's primary aim was to determine the age range at which each item is accomplished, utilizing the Malawi MDAT norms for the initial trial. Subsequent data collection will adopt the sequence based on the findings from the present validation study.” 

***9. Can the authors be clearer about how they obtained the sample of children for the study and if these children are representative of the general population in the Dominican Republic?***

******

 The children in this study attended CAIPIs and CAFIs in the metropolitan area. While this may not be a fully representative sample of the general population of children in the Dominican Republic, we are currently obtaining norms from a representative sample of INAIPI beneficiaries from all regions. Future data collection activities will also aim to include a sample of children outside of INAIPI to ensure greater representativeness. 

 How was this addressed in the manuscript? 

 We have included the following statement in the discussion section: “One limitation of this study is that the sample may not accurately reflect all children in the Dominican Republic since it comprises only those involved in INAIPI programs within the metropolitan region. During the study period, 77,000 children enrolled in INAIPI from the metropolitan region accounted for 1.23 percent of the total population of children ages 0 to 5. The limited sample size was influenced by the challenges posed by the COVID-19 pandemic, including restrictions and prevention measures.” 

***10. Were other demographic factors taken into account, such as birth weight, adversities, sociodemographic status, health status, frequency of admissions to hospital, nutritional status, etc.?***

 For the the current validation study, no other demographic factors were considered since we were looking for a progression in item order. However, future data collection activities, which will involve a larger sample of children from INAIPI, will take into account variables such as birth weight, prematurity, and other health factors. 

 How was this addressed in the manuscript?

 The following statement has been included in the method section. “No other demographic factors were considered for this analysis.” In addition, the following statement was included in the discussion section: “Another limitation is that the study did not account for other relevant factors in childhood development such as low birth weight and other health factors. However, future data collection activities, which will involve a larger sample of children from INAIPI, will consider variables such as birth weight, prematurity, and other health factors to determine cutoff points that better identify children at risk for developmental delay and to further understand the sensitivity of SIMEDID.”  

***11. The authors describe the use of Cronbach’s alpha for internal reliability and “split half parallel reliability”, but it is unclear if any reliability was done at the time of training and whether any quality control was done between examiners?***

 During the pilot (unpublished), Cronbach’s alpha and split-half parallel reliability were conducted to ensure the reliability of the instrument. Additionally, we conducted a quality control analysis to check for consistency between examiners. This involved comparing the mean scores of participants per evaluator.  

 How was this addressed in the manuscript?

 The following statement has been included in the method section: “To guarantee uniformity in assessments among evaluators, we conducted an analysis of variance (ANOVA), comparing the mean scores of participants within each age group per evaluator. The results revealed no significant differences 

 between evaluators within each age group across all dimensions.” 

***12. What did the 6-hour session for training consist of? Could it be clearer who did this and what is needed for the future? Were there online sessions? In-person sessions? Any materials that were created? Certification of assessors?***

 The 6-hour training session, conducted by INAIPI personnel, encompassed practical and theoretical components based on a module designed by experts in training and child development. The training, mainly conducted face-to-face, included virtual follow-up by the Child Development Evaluation Division. While there was no initial certification process for assessors, facilitators of the training received certification as official SIMEDID facilitators. The module covered essential topics such as the concept of child development, the Dominican Child Development Measurement System (SIMEDID), its structure, and dimensions. It explained who applies SIMEDID in INAIPI's service modalities and provided guidelines on application rules. Evaluators learned the assessment process in homes, including guidance for parents, the periodicity of the system, and the specifications of the tool. The practical section involved the hands-on use of evaluation kit materials in simulated assessments, emphasizing adherence to instructions for standardized data. The training concluded with a presentation on the app's functionality, using visual aids and videos to reinforce the covered content. 

 How was this addressed in the manuscript?

 The following statement has been included in the method section: “A total of 40 evaluators received a six-hour training session and conducted two practice evaluations. The training session comprised both theoretical and practical components. The training, facilitated by experts in training and child development, covered essential topics such as the concept of child development and SIMEDID. It included virtual follow-ups by the Child Development Evaluation Division and certification for facilitators. The module provided insights into SIMEDID's structure, dimensions, and its application in INAIPI's service modalities. Evaluators learned about assessment processes in homes, including guidance for parents, system periodicity, and tool specifications. The practical section involved hands-on use of evaluation kit materials in simulated assessments, emphasizing adherence to standardized data collection. The training concluded with a presentation on the SIMEDID app's functionality, utilizing visual aids and videos to reinforce the covered content. At the end of the training, evaluators were certified as official SIMEDID evaluators.”  

***13. Furthermore, could the authors provide more information about whether children were assessed with a disability, and if not, how did they decide to exclude them? Can the present tool go down further to assess a child who is not performing? How did the team decide if they had a child that they felt was below the norm?***

 For this specific data collection activity, we did not exclude children with disabilities because such data were not available. However, for the normalization study, in which we are setting the cutoff points to detect developmental delays, we have a mechanism to exclude them from the analysis, not from the evaluation. This way, we will be able to determine SIMEDID's sensitivity to disability. The tool is designed to assess children from ages 0 to 5, and there are items that can be used to assess children who are not performing at their age level. 

 How was this addressed in the manuscript?

 The following statement has been included in the discussion “Another limitation is that the study did not account for other relevant factors in childhood development, such as low birth weight and other health factors or disabilities. However, future data collection activities, which will involve a larger sample of children from INAIPI, will consider variables such as birth weight, prematurity, and other health factors to determine cutoff points that better identify children at risk for developmental delays and to further understand the sensitivity of SIMEDID.”  

***14. The authors describe that age did not predict certain items. Is this because these items are not acceptable or work in the setting, or is it because there were not enough children to assess these items? It seems to be the latter.***

 We did not have enough children to assess these items, and we are currently working on obtaining a sample of children within the age range to evaluate these items. We are meeting with other governmental institutions that work with infants younger than 45 days to expand the age range of the study. 

 How was this addressed in the manuscript?

 The following statement has been included in the discussion section: “We could not validate initial items of SIMEDID given that INAIPI does not provide services to infants younger than 45 days, given that mothers are on maternity leave during that period. However, the research team is meeting other governmental institutions that work with infants that young to expand the age range of the study. “ 

***15. It is not clear that the authors have definitively shown that the tool can sensitively identify changes. Could they make that clearer and that they have demonstrated its use in another population who are known to have a problem through doing a Gold Standard assessment of these children?***

 We have yet to demonstrate the tool's ability to sensitively identify changes, as this is the first large-scale data collection using this instrument. Our current focus is to establish the reliability and validity of the SIMEDID tool. However, we are designing a study to compare the SIMEDID tool with another widely used instrument by UNICEF that is applied during household surveys.   

 How was this addressed in the manuscript?

 The following statement has been included in the discussion section:  “We have yet to demonstrate the tool's ability to sensitively identify changes, as this is the first large-scale data collection using this instrument. However, since SIMEDID is already part of the monitoring strategy, with constant data collection, it would be relatively simple to demonstrate its sensitivity to changes.”  

***16. Could the authors provide a little more information on what they plan to do with the information from this study? At the moment, it seems like it goes into a data collection system, but what and how will they create “cut-offs” decided for referral? These next steps are very important and could be provided more clearly for the reader.***

 The information collected from this study will be used to establish cut-off scores for referral to early intervention services. We will use statistical methods to determine the optimal cut-offs.  A subsequent standardization study allowed us to establish correction parameters and normative scores, with which it will be possible to establish when alerts occur in the development of a child. 

 How was this addressed in the manuscript?  

  This was addressed in the Discussion section:  

 “These results will allow the pertinent institutions of the Dominican Republic to implement and report more accurate early childhood development indicators. Still, they will also contribute to creating a robust monitoring system with a high-quality data collection process that allows evidence-based and timely decision-making. Furthermore, such a system will contribute to generating longitudinal data that establish the association of childhood development with sociodemographic and psychosocial variables and determine the impact of initiatives and interventions ​(Richter et al., 2017)​, which is not sufficiently evaluated in most countries ​(Daelmans et al., 2017)​.” 
